# Microencapsulation of Rambutan Peel Extract by Spray Drying

**DOI:** 10.3390/foods9070899

**Published:** 2020-07-08

**Authors:** Luis Boyano-Orozco, Tzayhrí Gallardo-Velázquez, Ofelia Gabriela Meza-Márquez, Guillermo Osorio-Revilla

**Affiliations:** 1 Departamento de Ingeniería Bioquímica, Escuela Nacional de Ciencias Biológicas, Instituto Politécnico Nacional, Av. Wilfrido Massieu S/N, Col. Unidad Profesional Adolfo López Mateos, Zacatenco, Ciudad de México CP. 07738, Mexico; luisboyano8@gmail.com (L.B.-O.); ogmmz@yahoo.com.mx (O.G.M.-M.); 2 Departamento de Biofísica, Escuela Nacional de Ciencias Biológicas, Instituto Politécnico Nacional, Prolongación de Carpio y Plan de Ayala S/N. Col. Santo Tomás, Ciudad de México CP. 11340, Mexico; gtzayhri@yahoo.com

**Keywords:** antioxidant capacity, bioactive compounds, microencapsulation, rambutan, spray drying

## Abstract

Microencapsulation of bioactive compounds (BC) from rambutan peel by spray drying using DE10 maltodextrin as encapsulating agent was performed. The optimal conditions for the ethanolic extraction of BC were 60 °C, with a time of 1 h, 55% aqueous ethanol and three extraction cycles. The best spray drying encapsulating conditions for BC and antioxidant capacity (AC) were: inlet temperature 160 °C, outlet temperature 80 °C, and 10% encapsulating agent concentration in the feeding solution (core:encapsulating agent ratio of 1:4). With these conditions, retention and encapsulation efficiencies obtained were higher than 85%, the water activity value, moisture content and Hausner Index were of 0.25 ± 0.01, 3.95 ± 0.10%, and 1.42 ± 0.00, respectively. The optimized powder presented good solubility and morphological properties, showing microcapsules without ruptures. Based on these results, microencapsulation by spray drying is a viable technique which protects BC of rambutan peel, facilitating its application in the food, pharmaceutical, and cosmetic industries.

## 1. Introduction

In recent years, the interest of the consumer in food products rich in bioactive compounds (BC) has increased. This is because different research has related the consumption of these compounds with the prevention of different chronic diseases, among which, are the cardiovascular diseases [1]. To meet this demand, food manufacturers and researchers have focused on the development of products containing BC, attributing health benefits to these products [2]. Indeed, many plant extracts rich in BC are used as food supplements or are integrated into cosmetic and pharmaceutical formulations [3,4,5].

A good example of an extract rich in BC is the rambutan (*Nephelium lappaceum* L.) peel extract. Rambutan is an exotic tropical fruit that belongs to the Sapindaceae family. It is a native from Malaysia and Indonesia and is cultivated in some countries of Asia, Africa, and America [6,7]. Rambutan is generally consumed fresh, and, in the main producing countries, it is also preserved and processed in other products such as juices, jams, and jellies [8]. In the rambutan industrialization process, peels and seeds are the main waste which constitute almost 50% of the fruit weight [7]. Similarly, when it is consumed as a fresh fruit, people only eat the rambutan flesh and discard the peels and seeds.

Rambutan peels have attracted the attention of researchers due to their high content of BC, as: phenolics compounds (PC) and hydrolyzable tannins (HT) such as ellagic acid, corilagin, and geraniin, vitamin C, and some minerals (Cu, K, Fe, and Zn) [6,9,10]. Different researchers have studied the effect of the consumption of the rambutan peel extract on health, determining that this could be an important factor for the prevention of several diseases. In this sense, beneficial properties such as anti-inflammatory, antidiabetic, antioxidants, antiaging, antiobesity, antimicrobial, anti-osteoporosis, photoprotective, and anticancer have been reported for rambutan peel extract [6,11,12,13,14,15,16,17,18].

BC in general, and specifically from rambutan peel extract, are susceptible to degradation when they are exposed to different environmental conditions (light, oxygen, temperature, and moisture); in addition, the unsaturated bonds in their molecular structures could make them less stable [19,20]. Other difficulties can occur during their incorporation into any product such as its dark brown color, bitter taste, low solubility, reactions with other ingredients, low digestive stability, and low gastrointestinal absorption [21]. An alternative to solve these problems is microencapsulation, which apart from protecting the BC from degradation, can hide the bitter flavor and dark brown color of rambutan peel extract, facilitating its application in industrial products. Microencapsulation is a technology where a solid or liquid active material (core), can be coated with a continuous film (encapsulating agent or wall material) to preserve its stability and protect it from the environment [22]. The most used method to microencapsulate BC is spray drying, which is a rapid and low-cost process that can easily preserve heat-sensitive materials [23].

Recently, international organizations such as FAO have consider reducing food loss and waste as a priority [24]. Based on this, it is important to valorize the waste produced in the rambutan industrialization. Despite the multiple benefits that have been found in the rambutan peel extract, the protection of its BC by microencapsulation has not yet been reported.

The aim of this study was to take advantage of an agro-industrial residue rich in BC, by microencapsulating rambutan peel extract (*Nephelium lappaceum* L.) using spray drying with DE10 maltodextrin (MD) as an encapsulating agent and to evaluate the effects of the different operating conditions on BC content and physicochemical properties of the microcapsules. In this way, this study could be the starting point for the development of different functional products, food ingredients, and nutraceutical, pharmaceutical, and cosmetic products that can help to prevent diseases of great importance worldwide.

## 2. Materials and Methods

### 2.1. Materials

Rambutan fruits were obtained from a local market (Central de Abastos, Mexico City, Mexico). The encapsulating agent was maltodextrin (MD), with a dextrose equivalent (DE) of 10 (Amidex, Mexico City, Mexico). ABTS (2,2′-azino-bis-3-ethylbenzothiazoline-6-sulphonic acid), DPPH (2,2-diphenyl-1-picrylhydrazyl), and Trolox (6-hydroxy-2,5,7,8-tetramethylchroman-2-carboxylic acid) were purchased from Sigma-Aldrich (St. Louis, MO, USA); ethanol, methanol, potassium iodate, and tannic acid were obtained from Reasol (Mexico City, Mexico); potassium persulfate and gallic acid from Fermont (Mexico City, Mexico); Folin-Ciocalteu reagent from Meyer (Mexico City, Mexico); and sodium carbonate from Hycel (Mexico City, Mexico). All chemicals and solvents were analytical grade.

### 2.2. Preparation of Rambutan Peel for Extraction of Bioactive Compounds

The fruits were washed with water and soap, and the peels were separated manually and stored at −20 °C in ziploc bags until time of use. Peels were thawed at ambient temperature and cut into pieces of approximately 1 cm^2^. Then, they were cut into even smaller pieces in a food processor (MBR-1101, Magic Bullet, Los Angeles, CA, USA) during 20 s for further use in extraction.

### 2.3. Extraction of Bioactive Compounds from Fresh Rambutan Peel

The bioactive compounds from fresh rambutan peel were extracted by solid–liquid extraction using the conditions reported by Samuagam et al. [25]. Samples were mixed in a 1:10 sample:solvent ratio with 80% aqueous ethanol. Then, the mixture was kept for 2 h at 50 °C in an oven (OV-12 Lab Companion, Jeio Tech, Daejeon, Korea). The mixture was manually shaken for 20 s every 15 min. After two hours, the samples were centrifuged (MS-3400, Cole-Palmer, Vernon Hills, IL., USA) at 1796 g (3400 rpm) for 20 min. The supernatant was filtered through Whatman paper No. 6 (Whatman Ltd., Maidstoine, UK) and kept in an amber glass bottle until analysis. In order to quantify the total bioactive compounds (PC and HT) present in the rambutan peel, consecutive extraction cycles were performed, until the absorbance values during the determination of both PC and HT were less than 0.1. This was achieved with 5 consecutive extractions.

### 2.4. Drying of Rambutan Peel to Increase the Concentration of Bioactive Compounds in the Extract

In order to increase the concentration of BC in the extract for encapsulation purposes, the thawed rambutan peel pieces were dried at 45 °C for 24 h [9] in an oven and then milled in a food processor for 20 s and sieved in a 40 mesh sieve (420 µm). The material retained in the sieve was milled again for another 20 s and sieved. This process was repeated until most of the dried peel passed the sieve. The extraction process for BC in the dried peel powder was the same as described in Section 2.3. The effect of the drying process was evaluated by comparing the content of BC in the thawed and dried peels.

### 2.5. Number of Extractions Required to Extract at Least 90% of the Bioactive Compounds in Rambutan Peel

The number of extraction cycles (for extracting more than 90% of the BC) was determined from the 5 consecutive extractions performed to the dry rambutan peel and quantifying the BC and AC as response variables in each of the extracts. The extraction of both response variables was expressed as the cumulative percentage of the compounds in each extraction. The process was carried out in duplicate.

### 2.6. Optimization of Extraction of Bioactive Compounds from Rambutan Peel

For the optimization of the extraction of BC from the dried peel, a Box–Behnken experimental design (BBD) was used, studying 3 factors at 3 levels: solvent concentration (aqueous ethanol: 55, 75, and 95% *w/w*), extraction temperature (40, 50, and 60 °C), and extraction time (1, 2, and 3 h). The experiment consisted of 30 runs including three central points to achieve the maximum amount of phenolic compounds (PC), hydrolyzable tannins (HT), and antioxidant capacity (AC) in the extraction process.

### 2.7. Preparation of the Solution for the Spray Drying Process

Prior to the preparation of the solution of rambutan peel extract and the maltodextrin used as encapsulating agent, it was necessary to evaporate the ethanol present in the extract, which was performed using a rotavapor (Rotavapor^®^ R-300, BUCHI, Flawil, Switzerland) monitoring the boiling temperature of the solution. This was necessary to avoid precipitation of maltodextrin in the solution to be dried. A 30% *w/w* solution of MD was prepared and left to stand overnight to eliminate air bubbles. The MD solution was then mixed with the concentrated rambutan extract to obtain final concentrations of 10 and 13% of encapsulating agent in the solution to be dried. This corresponded to a core (dry solids of extract):encapsulating agent (solids of MD) ratio of 1:4 and 1:6, respectively. The concentration of the encapsulating agent was selected based on preliminary experiments. The quantities used of MD and rambutan extract in preparing the solutions were calculated with a mass balance.

### 2.8. Microencapsulation by Spray Drying of the Rambutan Peel Extract

The microencapsulation of the rambutan peel extract was accomplished with a semi-pilot spray dryer (Mobile Minor MM, GEA, Gladsaxe, Denmark) equipped with a spray two-fluid nozzle. For this process, a 2^3^ factorial design was applied, studying the effect of the variables: inlet air temperature (160 and 180 °C), outlet air temperature (70 and 80 °C), and encapsulating agent concentration (10 and 13% in the solution to be dried) on the following response variables: PC, HT, and AC retention and encapsulation efficiencies; water activity (a_w_); moisture content; and Hausner ratio. All treatments were performed in duplicate. Powders were stored in sealed amber glass bottles and kept at −20 °C until analysis.

#### Analysis of Powders

The powders obtained in the different drying conditions were reconstituted in water at the same solids concentration as in the initial solution before spray drying. The reconstituted powders were subjected to the same analysis procedure used in the solution to be dried.

### 2.9. Analytical Methods

#### 2.9.1. Total Phenolic Compounds Determination

Total PCs were determined according to the method described by Singleton and Rossi [26]. For the analysis, 0.5 mL of each sample was mixed with 7.5 mL of distilled water, followed by the addition of a 50% (*v/v*) Folin–Ciocalteu reagent. After 8 min, 1.5 mL of sodium carbonate (20% *w/w*) was added and left to stand for 60 min in the dark. The absorbance of the solution was measured at 750 nm in a spectrophotometer (Jenway 320D, Staffordshire, UK) and the PC were quantified using a gallic acid standard calibration curve (Absorbance = 9.9301(mg GAE) − 0.0017). Results were expressed as milligrams of gallic acid equivalents per gram of extract (wet weight) (mg GAE/g_ext_) for the extraction optimization and as milligrams of gallic acid equivalents per gram of dry weight (mg GAE/g_dw_) for the rambutan peel characterization, the solution to be dried, and the reconstituted powders.

#### 2.9.2. Antioxidant Capacity Determination

AC was measured using two radical scavenging methods: the ABTS [27] and DPPH [28] radical inhibition assays. For both methods, the encapsulating agent (MD) was previously precipitated with ethanol (ABTS) or methanol (DPPH) in the samples of the solutions to be dried and the reconstituted powders. This precipitation was necessary since the encapsulating agent is insoluble in such solvents, which are used for the preparation of the radicals. After the precipitation, the solution was centrifuged at 1796 g (3400 rpm) during 5 min and filtered through a glass microfiber filter paper with 0.6 µm of pore size (ADVANTEC, Tokyo, Japan) [29]. Aliquots of the filtrate were used for the analyses. For ABTS, the prepared radical was diluted with ethanol to obtain an absorbance of 0.7 (±0.05) at 734 nm. Subsequently, 3 mL of the ABTS radical was added to 0.3 mL of the sample, and vortexing for 6 min. Finally, the absorbance was monitored at 734 nm. For the DPPH radical inhibition assay, 3.9 mL of the prepared radical was added to 0.1 mL of the sample, and vortexing for 10 s. The mixture was allowed to stand for 1 h in the dark at room temperature. The decrease in absorbance was measured at 515 nm.

The antioxidant value of the samples was determined, for both methods, using the inhibition percentage (%) (Equation (1)) and a Trolox standard calibration curve. Results were expressed as micromoles of Trolox equivalents per gram of extract (wet weight) (µmol TE/g_ext_) for the extraction optimization and micromoles of Trolox equivalents per gram of dry weight (µmol TE/g_dw_) for the rambutan peel characterization, the solution to be dried, and the reconstituted powders:

(1) $$\%Inhibition=\left(\frac{Control\;Abs-Sample\;Abs}{Control\;Abs}\right)\times 100$$

where Abs: absorbance.

#### 2.9.3. Hydrolyzable Tannins Determination

HT were quantified as described by Çam and Hisil [30]. Briefly, 1 mL of each sample was placed in separate test tubes, adding 5 mL of 2.5% (*w/w*) potassium iodate. The mixture was shaken during 10 s and after 4 min the absorbance was measured at 550 nm in a spectrophotometer. A tannic acid calibration curve (Absorbance = 0.2796(mg TAE) + 0.013) was used to quantify HT. Results were expressed as milligrams of tannic acid equivalents per gram of extract (wet weight) (mg TAE/g_ext_) for the extraction optimization and milligrams of tannic acid equivalents per gram of dry weight (mg TAE/g_dw_) for the rambutan peel characterization, the solution to be dried, and the reconstituted powders.

#### 2.9.4. Determination of Bioactive Compounds on the Surface of the Particles

The BC on the surface of the particles was determined according to the method described by Robert et al. [31]. The powder was mixed with an ethanol:methanol (1:1) solution and vortexed for 1 min. The mixture was filtered through a glass microfiber filter paper with 0.6 µm of pore size. The filtrate was analyzed to determine PC and HT.

#### 2.9.5. Determination of Retention and Encapsulation Efficiency

Retention efficiency (RE) was calculated by determining the BC content in the solutions to be dried (extract + encapsulating agent) and in the reconstituted powders, expressing the result in percentage (Equation (2)):

(2) $$RE=\left(\frac{BC\;content\;in\;powder/g_{dw}}{BC\;content\;in\;solution\;to\;be\;dried/g_{dw}}\right)\times 100$$

Encapsulation efficiency (EE) was calculated by determining the difference between the BC content retained in the powder and the BC content on the surface of the particles, divided by the BC content in the solution to be dried (Equation (3)):

(3) $$EE=\left(\frac{BC\;content\;in\;powder-BC\;content\;on\;surface\;particles/g_{dw}}{BC\;content\;in\;solution\;to\;be\;dried/g_{dw}}\right)\times 100$$

where BC: Bioactive compounds

#### 2.9.6. Determination of Moisture Content and Water Activity (a_w_)

Moisture content was determined using a thermobalance (OHAUS MB200, Montville, NJ, USA), placing 0.5 g of the sample at 110 °C until there was no change in weight greater than 0.01 g in 90 s. Water activity (a_w_) was measured using an AquaLab Cx-2 (Decagon, Pullman, WA, USA). Both determinations were performed in triplicate.

#### 2.9.7. Bulk Density Determination

Bulk density was measured by weighing 1 g of the powder sample and placing it slowly (without allowing compaction) in a 10 mL graduated cylinder. The volume occupied by the sample was registered. Bulk density was calculated as the ratio between the mass of the powder contained in the cylinder and the volume occupied [32].

#### 2.9.8. Tapped Density Determination

Tapped density was determined by weighing 1 g of sample and placing it in a 10 mL graduated cylinder. The cylinder was tapped on a flat surface to reach a constant volume. The tapped density was calculated as the ratio between the mass of powder contained in the cylinder and the volume occupied [33].

#### 2.9.9. Hausner Ratio Determination

Hausner ratio was calculated as the ratio between the tapped density and the bulk density (Equation (4)) [34]:

(4) $$Hausner\;Ratio=\frac{Tapped\;density\;}{Bulk\;density\;}$$

### 2.10. Characterization of the Powder Obtained under Optimal Spray Drying Conditions

The powder obtained under optimal spray drying conditions was subjected to solubility, particle size distribution, and surface morphology determinations.

#### 2.10.1. Solubility Determination

Solubility was determined according to the method described by Alvarez-Gaona et al. [35] with slight modifications. In addition, 1 g of powder was dissolved at ambient temperature (25–28 °C) in 50 mL of distilled water by stirring during 5 min with a magnetic stirrer. Then, the solution was centrifuged at 1796× *g* (3400 rpm) during 5 min. Finally, 25 mL of the supernatant was dried in an oven for 5 h at 105 °C, determining the percentage of powder that was dissolved.

#### 2.10.2. Particle Size Distribution Determination

Particle size distribution was measured using a laser diffraction particle size analyzer (IM 026 2006 series, Malvern, UK). A small amount of powder was dispersed in hexane (REASOL, Mexico City, Mexico) and the particle size distribution, span, equivalent spherical diameter (D[4,3]), and Sauter diameter (D[3,2]) were determined using a 100 mm lens.

#### 2.10.3. Surface Morphology Determination

The surface morphology of the particle was observed with a scanning electron microscope (SEM) (JSM-5800LV, Jeol, Peabody, MA, USA) set to an acceleration voltage of 10 kV. Microcapsules were placed in double-faced adhesive tape stubs, then coated with gold, and finally observed using a 500 and 1000× magnification.

### 2.11. Statistical Analysis

Results were expressed as the mean value ± standard deviation. Significant differences between means were determined by analysis of variance (ANOVA) using Minitab statistical software, version 17 (Minitab Inc., State College, PA, USA).

## 3. Results and Discussion

### 3.1. Bioactive Compounds in Fresh Rambutan Peel

Table 1 shows the results obtained for the determination of the BC in fresh rambutan peel. The PC content was similar to the content for rambutan peel reported by Gusman and Tsai (298.47 ± 2.10 mg GAE/g_dw_) [36]. The values of the PC found in rambutan peel in the present study were considerably higher than the values of PC (mg GAE/g_dw_) reported for other tropical fruits by-products materials, like: pineapple (27.87), cashew apple (65.88), guava (19.87), surinam cherry (126.96), and sapodilla (10.53), among others [1]. Regarding the HT content, most of the published data on HT of rambutan peel, report the different fractions of HT determined by HPLC (ellagic acid, corilagin, and geraniin), rather than the total HT content; because of that, the values shown in Table 1 were compared with the HT content of pomegranate peel which is one of the by-products with the highest content of these compounds. As can be seen in Table 1, HT of rambutan peel was almost twice that for pomegranate peel (262.7 mg TAE/g_dw_) reported by Çam and Hisil [30]. The AC content, shown in Table 1, were higher than the values for rambutan peel (667.90 μmol TE/g_dw_) reported by Chunglok et al. [37], which may be due to differences in extraction methods, type, and concentration of chemical components, which in turn depend on various environmental conditions, and therefore to the geographical origin of the samples. Based on these results, rambutan peel can be considered as a by-product with a high content of BC with a potential use in the food, pharmaceutical, and cosmetic industries.

### 3.2. Comparison between the Amount of Bioactive Compounds of Fresh and Dry Rambutan Peel

As mentioned before, rambutan peel was dried in order to increase the concentration of BC in the extract. The effect of this drying process was assessed by comparing the BC content in the dried and fresh peels. After the drying process, PC decreased 10.52% while HT decreased 39.20%. Similar to PC, AC decreased 7.45% for the ABTS radical and 13.02% for the DPPH radical. PC may be primarily responsible for the AC present in rambutan peel since the drying process similarly affected both parameters [38]. The losses of HT could be due to hydrolysis and spontaneous rearrangement of the hexahydroxydiphenoyl (HHDP) and dehydrohexahydrodiphenoyl (DHHDP) groups to yield water insoluble bislactone ellagic acids, during exposure to drying temperature [39].

### 3.3. Optimization of Extraction Parameters for Bioactive Compounds from Rambutan Peel

**Figure 1:**
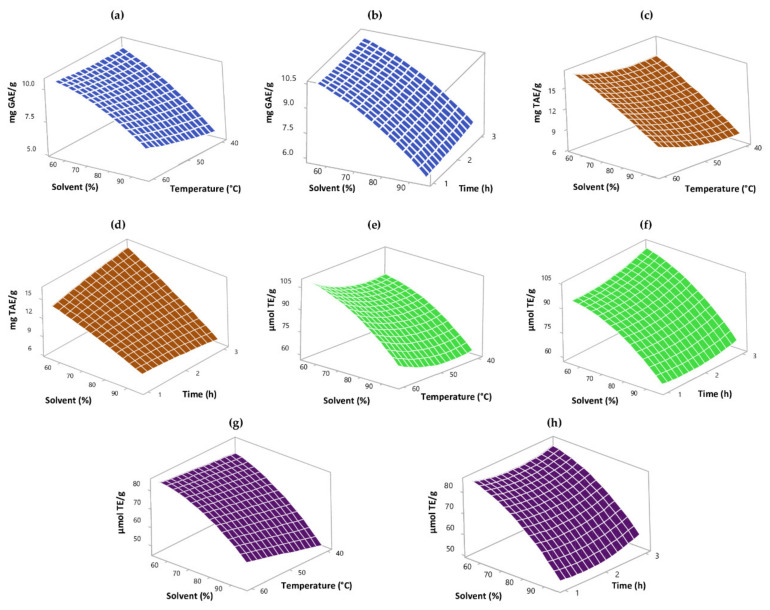
Three-dimensional surface plot of the effect of process variables on the extraction of PC (**a**,**b**) and HT (**c**,**d**), and the AC obtained, quantified by ABTS (**e**,**f**) and DPPH (**g**,**h**).

From the five consecutive extractions performed to the dried rambutan peel, in the conditions mentioned in Section 2.3, three extraction cycles were selected. With this number of extractions, 95.8% of PC and 92.13% of HT were extracted, as well as 97.12% (ABTS) and 99.34% (DPPH) of AC. The effect of the process variables on the extraction of BC of rambutan peels was studied through an ANOVA. According to this analysis, there was a significant effect (*p* < 0.05) of temperature, ethanol concentration, and the interaction of these variables on the extraction of PC. The surface plot in Figure 1a indicates that the highest temperature (60 °C) and lower ethanol concentration (55%) favored PC extraction. On the other hand, time did not affect (*p* < 0.05) the extraction of PC (Figure 1b). Maran et al. [40] explain that, by increasing extraction temperature (40 to 50 °C), the vapor pressure of solutes also increases; this improves PC solubility, therefore their extraction. This can explain the results of the present study.

In the case of HT, as occurred for PC, according to the ANOVA, temperature and ethanol concentration presented a significant effect (*p* < 0.05) on their extraction. The variable time did not affect (*p* < 0.05) the extraction of HT; nevertheless, its interaction with ethanol concentration did, increasing the extraction at lower ethanol concentration and higher extraction time. Figure 1c,d illustrates that raising the extraction temperature and decreasing ethanol concentration positively affected HT extraction. Regarding AC quantified by the ABTS method, all three studied variables had a significant effect (*p* < 0.05). Figure 1e shows that increasing the extraction temperature and reducing ethanol concentration (55%) favored the AC obtained. Figure 1f illustrates that AC quantified by ABTS was significantly affected by time (*p* < 0.05), increasing with it. In the case of the AC determined by the DPPH method, only the temperature and solvent concentration variables had a significant effect (*p* < 0.05) (Figure 1g,h). It showed the same trend of AC by ABTS, increasing with higher extraction temperature (60 °C) and lower ethanol concentration (55%). It has been reported that there is a high correlation between the PC and AC present in some plant extracts like rambutan peel extract [38]. In this sense, if high extraction temperatures increased PC extraction, it might increase AC, as it happened in the present work.

### 3.4. Optimum Extraction Conditions

Using Minitab version 17, a Box–Behnken design was performed to achieve the maximum content of all variables in the extraction process. The optimum extraction conditions were estimated using the desirability function, and the results were experimentally validated. The optimum levels of the variables were: extraction temperature 60 °C, extraction time 1 h, and ethanol concentration 55%, with three consecutive extractions. Predicted values were: PC: 10.82 mg GAE/g_ext_, HT: 15.48 mg TAE/g_ext_, AC (ABTS): 105.53 µmol TE/g_ext_ and AC (DPPH): 87.77 µmol TE/g_ext_ with a desirability value (D) of 0.9139. The results obtained in the experimental validation of predicted values were: 10.85 ± 0.34 mg GAE/g_ext_, 17.80 ± 0.18 mg TAE/g_ext_, 103.11 ± 1.60 µmol TE/g_ext_ and 89.75 ± 3.71 µmol TE/g_ext_, respectively. As can be seen, theoretical and experimental results obtained using optimum conditions were found to be similar.

### 3.5. Microencapsulation by Spray Drying

#### 3.5.1. Retention Efficiency of Phenolic Compounds

**Figure 2:**
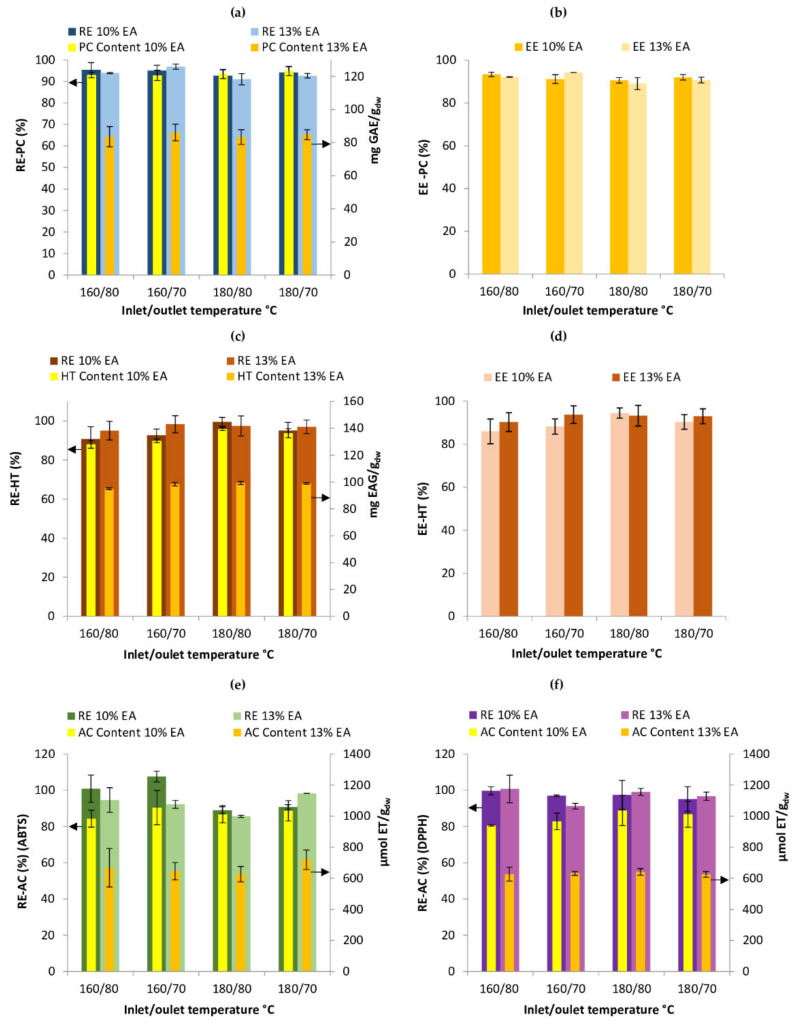
Concentration, retention and encapsulation efficiency of bioactive compounds (BC) in microcapsules. (**a**) content and retention efficiency of PC; (**b**) encapsulation efficiency of PC; (**c**) content and retention efficiency of HT; (**d**) encapsulation efficiency of HT; (**e**) content and retention efficiency of AC (ABTS); (**f**) content and retention efficiency of AC (DPPH). Data are expressed as the mean ± standard deviation of the analysis in duplicate drying runs. The arrows indicate the axis to which the bars belong; RE: retention efficiency; EE: encapsulation efficiency; EA: encapsulating agents; PC: phenolic compounds; HT: hydrolyzable tannins; AC: antioxidant capacity.

The retention efficiency (RE) of PC in the microcapsules, shown in Figure 2a, varied from 92.72 ± 2.63 to 95.40 ± 3.31% and 91.01 ± 2.64 to 96.82 ± 1.19% for 10 and 13% encapsulating agent concentration in the drying solution, respectively. Similar PC retention has previously been reported for the spray drying of grape skin extract (81.4–95.3%) [41]. Based on the statistical analysis, the inlet air temperature had a significant effect (*p* < 0.05) on the PC retention; however, this effect was negative, meaning that a higher inlet air temperature resulted in a lower retention efficiency. The outlet temperature and encapsulating agent concentration did not have a significant effect (*p* < 0.05). In the spray drying process, several factors can generate the degradation of PC, among these can be mentioned the exposure to oxygen and high temperatures [41]. Nevertheless, it has been reported that high inlet air temperature leads to the rapid formation of semipermeable membrane on the surface of the drop, which provides optimal retention of the core material (PC) [42], as it happened in the present work.

As shown in Figure 2a, the amount of PC ranged from 120.70 ± 3.08 to 123.30 ± 2.77 mg GAE/g_dw_ when the encapsulating agent concentration was 10% and 83.35 ± 4.40 to 86.15 ± 5.0 mg GAE/g_dw_ when it was 13% in the solution to be dried. Similar values were found by Çam et al. [43] in the spray drying of pomegranate extract using MD as encapsulating agent. PC content decreased as the encapsulating agent concentration increased in the solution to be dried, due to a dilution effect, thus, obtaining less PC in powders with 13% MD (Figure 2a). Despite the drying conditions and the encapsulating agent concentration used, PC retention was above 90% for all the samples. In this sense, powders obtained with 10% MD have the advantage of presenting higher PC content (mg GAE/g_dw_) than powders obtained with 13% MD.

#### 3.5.2. Encapsulation Efficiency of Phenolic Compounds

As can be observed in Figure 2b, the encapsulation efficiency (EE) of PC varied from 90.58 ± 1.27 to 93.40 ± 1.04% and from 89.07 ± 2.79 to 94.26 ± 0.01% for runs with 10 and 13% encapsulating agent concentration, respectively. These results are higher than those reported for encapsulation of pomegranate extract using MD as encapsulated agent (69.80%) [42] and green coffee extract (84 ± 2.6%) [44] with a core:encapsulating agent ratio of 1:9 and 1:1, respectively.

It has been reported that the more encapsulated the agent the better encapsulation efficiencies could be obtained; however, a lower amount of encapsulating agent could help to decrease the costs of the drying process, and to obtain more amount of BC per g of capsules. In the present study, the core:encapsulating agent ratio of 1:4 and 1:6 were used seeking a balance of process costs and the protective effect of the encapsulating agent. The statistical analysis indicated that the different encapsulating conditions did not influence the encapsulation efficiency significantly. In this sense, regardless of the processing condition, good protection of the PC would be expected since all runs showed encapsulation efficiencies greater than 89%.

#### 3.5.3. Retention and Encapsulation Efficiency of Hydrolyzable Tannins

The combined effects of drying temperatures and encapsulating agent concentration on the content, retention, and encapsulation efficiencies of HT are presented in Figure 2c,d. The HT content in the capsules varied from 95.10 ± 0.70 to 139.48 ± 1.19 TAE/g_dw_. As shown in Figure 2c, the retention efficiencies of HT were above 90% for all conditions. In the same way, the encapsulation efficiencies of these compounds presented values in the powders, above 85% in all drying conditions (Figure 2d). The statistical analysis did not show significant difference (*p* < 0.05) among all the drying conditions for these variables. These results indicate that, no matter the drying condition, there will be high efficiencies of both retention and encapsulation of HT. Nevertheless, the samples with 10% encapsulating agent have higher HT content per g of capsules than with 13% MD.

#### 3.5.4. Retention Efficiency of Antioxidant Capacity

Figure 2e shows that the AC of capsules varied between 983.48 ± 54.48 to 1054.55 ± 110.58 and 626.52 ± 49.36 to 719.5 ± 62.82 µmol TE/g_dw_ for 10 and 13% encapsulating agent concentration, respectively for the ABTS method. For the DPPH method (Figure 2f), it varied in the range of 938.99 ± 4.69 to 1036.13 ± 96.50 µmol TE/g_dw_ when the encapsulating agent concentration was 10% and from 626.50 ± 44.88 to 641.02 ± 21.51 when it was 13%. Results showed that AC content decreased with the encapsulating agent concentration due to the dilution effect (increase in the total solid content of the feed).

As shown in Figure 2e,f, most of the antioxidant capacity of the solution to be dried was retained in the capsules, regardless the method used to determine it. The AC retention efficiency measured with ABTS ranged from 89.00 ± 1.93 to 107.58 ± 3.00% using 10% MD as an encapsulating agent. These values were similar to those obtained with 13% (85.57 ± 0.63 to 98.26 ± 0.07%). The statistical analysis showed that temperatures had a significant negative effect (*p* < 0.05) on the retention efficiency of AC with ABTS. In the case of AC determined by DPPH, there was not significant effect (*p* < 0.05). However, the retention efficiencies were similar to those obtained with ABTS (95.13 ± 6.83 to 99.71 ± 2.21% for 10% of encapsulating agent and 91.22 ± 1.57 to 100.71 ± 7.62% for 13%). The high retention efficiencies of AC in the capsules for the different operating conditions might be related to the high retention efficiencies of PC. According to these results, changing the encapsulating agent concentration from 10 to 13% in the solution to be dried does not affect the retention and encapsulation of the BC and AC in the powder. Nevertheless, it affects the amount of these compounds per g_dw_ because of the dilution effect.

### 3.6. Physicochemical Properties of Encapsulated Powders

Table 2 shows that the powders obtained in the different drying conditions had a moisture content from 3.42 ± 0.11 to 5.39 ± 0.27%. Moisture content was significantly affected (*p* < 0.05) by inlet and outlet temperatures. In this sense, with a lower inlet temperature and a higher outlet temperature, the moisture of the powders was lower. The concentration of the encapsulating agent did not present a significant effect (*p* < 0.05). It has been reported that an increase in the inlet temperature leads to an increase in the outlet temperature, but also a decrease in moisture of the powder [45]. The moisture content of the powders is typical for spray dried products.

The water activity a_w_ is a parameter that indicates stability of a food product. Several authors have reported that a_w_ values below 0.6 indicate that the product is stable at the microbiological and chemical level because of the low amount of free water available for biochemical reactions to occur [46]. The values of a_w_ obtained in the powder ranged from 0.305 ± 0.02 to 0.381 ± 0.04 for all operating conditions. According to the statistical analysis, a_w_ was affected significantly by the outlet temperature (*p* < 0.05), but as can be seen in Table 2, a_w_ was always below 0.390. In this study, considering the low values of a_w_ and moisture content of the powder for all operating conditions, good stability of these could be expected.

As shown in Table 2, the bulk density of the powders obtained in all operating conditions presented values of 0.195 ± 0.01 to 0.251 ± 0.00 g/mL. According to the statistical analysis, the outlet temperature affected this variable significantly (*p* < 0.05), showing higher values of bulk density with higher outlet temperature. Nevertheless, the bulk density for all drying conditions was around 0.22 g/mL. Factors like the distribution and particle size affect bulk density, since smaller particles could occupy the space among the largest, generating an increase in density [47].

In the determination of tapped density, the spaces between the particles are reduced being impacted by an external force [48]. In this study, the tapped density ranged from 0.286 ± 0.02 to 0.320 ± 0.01 g/mL (Table 2).

The powder’s flowability was evaluated through the Hausner ratio. The Hausner ratio is an indicator of cohesion of powder, and it was calculated using the bulk and tapped density values. Table 2 shows that values of the Hausner ratio obtained in powders varied from 1.24 ± 0.15 to 1.45 ± 0.15. The statistical analysis showed that none of the variables had a significant effect (*p* < 0.05) on Hausner ratio. These results indicate poor flow properties of the powders due to their high cohesiveness, as reported by Ortiz-Basurto et al. [33].

### 3.7. Optimized Spray Drying Conditions

The optimization of the spray drying process was carried out considering the variables which were significantly affected by operating conditions in the 2^3^ factorial design. It was performed to achieve the maximum retention efficiency of PC and AC (ABTS) and minimum moisture content and a_w_. The predicted optimum conditions obtained were: 160 °C inlet air temperature, 80 °C outlet air temperature and 10% MD concentration. An experimental verification was performed in duplicate dryings using the predicted optimum conditions. Table 3 shows the predicted and experimental values of the encapsulation response variables in the optimum operating condition. All studied variables, even those that were not affected by the operating conditions, were taken into account for this comparison.

As it is observed in Table 3, the experimental values were similar to the predicted ones, confirming the effectiveness of the model in predicting the concentrations of BC and physicochemical properties in powders.

### 3.8. Properties of the Optimized Encapsulated Powder

After the selection and validation of the best spray drying conditions, the percentage of solubility, particle size, and the surface morphology of microcapsules were determined.

#### 3.8.1. Solubility

In the present study, the water solubility of the powders obtained in the best spray drying conditions was 98.65 ± 0.05%. This indicates that compounds in rambutan peel are highly soluble in water, which suggests that the powder product could be added to various products in the food industry.

#### 3.8.2. Particle Size Distribution

**Figure 3:**
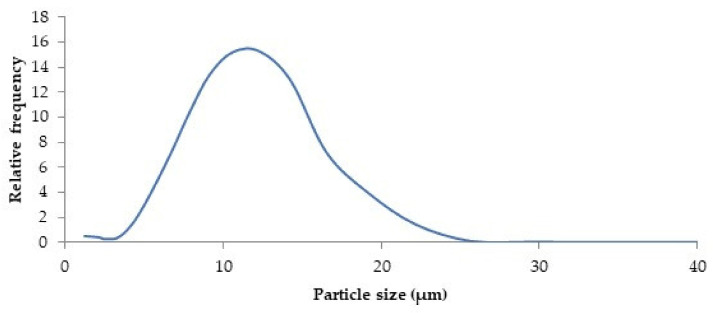
Particle size distribution of powder microcapsules obtained in the optimum spray drying conditions of rambutan peel extract (Ti 160 °C; To 80 °C; 10% MD).

The particle size distribution of powders in the optimum drying conditions is shown in Figure 3.

As can be seen in Figure 3, a monomodal, homogeneous, and narrow distribution was obtained, with particle size ranging from 1.215 to 29.75 μm and a span value of 0.96 ± 0.01. According to Moreno et al. [45], the closer the span value is to 1, the narrower the particle size distribution. Sauter diameter (D[3,2]) and equivalent spherical diameter (D[4,3]) were determined, obtaining 8.29 ± 0.03 and 10.18 ± 0.12 μm, respectively.

The particle size is one of the most important parameters that must be considered in the application of the spray-dried product in food processing. Based on Gómez-Aldapa et al. [49], particle sizes of 10 μm or less can contribute to reducing the impact on texture when incorporated into a food model system.

#### 3.8.3. Surface Morphology of Microcapsules

**Figure 4:**
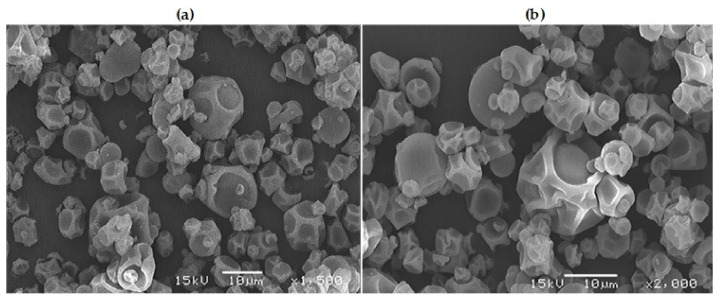
Image of microcapsules under the scanning electron microscope of rambutan peel extract microencapsulated by spray-drying (Ti 160 °C; To 80 °C; 10% MD). (**a**) 1500× magnification, (**b**) 2000× magnification, all at 15 kV.

Images of the particle surface morphology obtained through scanning electron microscopy (SEM) of the encapsulated powder are shown in Figure 4.

The micrographs on Figure 4 show microcapsules with a shrunken spherical form (dented surface). Particles of various sizes are present, having in general microcapsules with a size of 8–10 μm approximately. This is consistent with the particle size distribution reported in Section 3.8.2. As seen in Figure 4, very few microcapsules presented ruptures or cracks, which is convenient for the protection of the BC. During the spray drying process, the morphological irregularities in the powders are attributed to the water evaporation rate. Indeed, with the use of higher drying temperature (faster evaporation), it will have smoother and more defined surfaces [50]. On the other hand, if the temperature is not so high, the particles suffer a shrinkage during the process of moisture evaporation [50], as happened in the present work.

## 4. Conclusions

The present work shows that rambutan peels are rich in PC, HT, and AC, so these agroindustrial waste have a high biological value and great potential to be used as a source of natural antioxidants. The extraction process of BC from rambutan peel was successfully optimized using a Box–Behnken design. The optimal extraction conditions were: three consecutive extractions at 60 °C, extraction time 1 h and aqueous ethanol concentration of 55%. During the microencapsulation of rambutan peel extract with MD DE10, by the spray drying, the encapsulating agent concentration had no effect on any of the response variables. Nevertheless, with a concentration of 10% of the encapsulating agent (equivalent to a core:encapsulating agent ratio of 1:4), the powders presented a greater amount of BC per g of microcapsules than the powders with 13% encapsulating agent (equivalent to a core:encapsulating agent ratio of 1:6). This suggests a more economical process because less encapsulating agent is needed. Drying temperatures had significant effects on PC and AC retention (ABTS), moisture, and water activity of the powder obtained by spray drying.

An optimization of the drying conditions based on the maximum values of PC and AC retentions and minimum values of moisture and water activity, indicated that the best spray drying conditions were: an inlet temperature of 160 °C, outlet temperature of 80 °C, and a concentration of encapsulating agent of 10%. These optimized conditions provided the highest retention and encapsulation efficiencies (>85%) of the BC and AC, with satisfactory physicochemical properties that facilitate its stability and possible application of the microcapsules. Microencapsulating the BC of the rambutan peel is an excellent option to extend its shelf life and present them in a powdered product that can be used as an ingredient rich in natural antioxidants for the food, pharmaceutical, and cosmetic industry. In the food and pharmaceutical industries, encapsulated rambutan peel extract can be used to help prevent different diseases. It can be incorporated into food matrices (for example, yogurt or jellies), offered as food supplements, or included in cosmetic and personal care products such as shower and bath gels, lotions, and creams. Future studies (in vivo) are needed to confirm the biological activities of the powder.

## Figures and Tables

**Table 1:** Bioactive compounds in fresh rambutan peel.

Determination	Result
Phenolics compounds (PC)	304.52 ± 17.52
Hydrolyzable tannins (HT)	578.22 ± 11.37
Antioxidant capacity (ABTS)(AC)	2935.31 ± 161.52
Antioxidant capacity (DPPH)(AC)	2490.16 ± 187.70
Moisture content (%)	78.13 ± 0.01

Results are expressed as the mean ± standard deviation, *n* = 3. PC: mg GAE/g_dw_, HT: mg TAE/g_dw_, AC: μmol TE/g_dw_.

**Table 2:** Physicochemical properties of encapsulated powders.

Operating Conditions (Ti-To-EA)	Moisture (%)	Water Activity (a_w_)	Bulk Density (g/mL)	Tapped Density (g/mL)	Hausner Ratio
160-80-10	3.59 ± 0.20	0.327 ± 0.03	0.195 ± 0.01	0.286 ± 0.02	1.45 ± 0.15
160-70-10	5.08 ± 0.23	0.355 ± 0.03	0.220 ± 0.02	0.287 ± 0.02	1.30 ± 0.03
180-80-10	4.24 ± 0.17	0.319 ± 0.01	0.225 ± 0.01	0.286 ± 0.02	1.28 ± 0.18
180-70-10	5.36 ± 0.45	0.337 ± 0.02	0.238 ± 0.01	0.309 ± 0.02	1.29 ± 0.03
160-80-13	3.42 ± 0.11	0.305 ± 0.02	0.227 ± 0.01	0.289 ± 0.03	1.27 ± 0.08
160-70-13	5.08 ± 0.32	0.332 ± 0.03	0.251 ± 0.00	0.311 ± 0.03	1.24 ± 0.15
180-80-13	4.19 ± 0.29	0.331 ± 0.00	0.231 ± 0.01	0.320 ± 0.01	1.39 ± 0.04
180-70-13	5.39 ± 0.27	0.381 ± 0.04	0.237 ± 0.01	0.311 ± 0.01	1.32 ± 0.02

Results are expressed as the mean ± standard deviation of the analysis in duplicate drying runs. Ti: inlet temperature; To: outlet temperature; EA: % encapsulating agent.

**Table 3:** Experimental and predicted values for all variable studied for the encapsulated powder of rambutan peel extract obtained from the 2^3^ factorial design.

Response Variable	Predicted Values	Experimental Values
PC Retention (%)	95.40	97.72 ± 2.47
PC Encapsulation (%)	93.40	96.56 ± 2.47
AC Retention (ABTS) (%)	100.86	98.50 ± 2.79
AC Retention (DPPH) (%)	95.15	102.31 ± 0.59
HT Retention (%)	90.81	90.70 ± 2.53
HT Encapsulation (%)	85.91	86.10 ± 2.47
Water Activity (a_w_)	0.33	0.25 ± 0.01
Moisture (%)	3.59	3.95 ± 0.10
Hausner ratio	1.35	1.42 ± 0.00

Experimental values are expressed as the mean ± standard deviation of the analysis in duplicate drying runs. PC: phenolics compounds, HT: hydrolyzable tannins, AC: antioxidant capacity.
